# Off‐midline versus supraumbilical specimen extraction sites in robot‐assisted radical prostatectomy

**DOI:** 10.1111/bju.70400

**Published:** 2026-08-05

**Authors:** Jonathan Vollemaere, Julius Bastian, Jan Mink, Gudrun Wagenpfeil, Martin Janssen, Christina Maßmann, Matthias Saar, Stefan Siemer, Michael Stöckle, Julia Heinzelbecker

**Affiliations:** ^1^ Department of Urology and Pediatric Urology University Medical Centre Saarland and Saarland University Homburg/Saar Germany; ^2^ Institute of Medical Biometry, Epidemiology and Medical Informatics Saarland University Campus Homburg/Saar Homburg/Saar Germany; ^3^ Present address: Algemeen Ziekenhuis Sint‐Maria Halle, Halle, Flemish Brabant Ghent University Ghent Belgium; ^4^ Present address: Orthopaedic‐Surgical Doctor's Office St. Wendel Germany; ^5^ Present address: Department of Urology, Faculty of Medicine Medical Centre of the University of Münster Münster Germany; ^6^ Present address: Department of Urology, Sindelfingen Medical Center University of Tuebingen Tuebingen Germany; ^7^ Present address: Department of Urology and Pediatric Urology University Hospital RWTH Aachen Aachen Germany; ^8^ Present address: Department of Urology University Medical Center Marburg and Marburg University Marburg Germany

**Keywords:** incisional hernia, robot‐assisted radical prostatectomy, prostate cancer, incidence, extraction site

## Abstract

**Objectives:**

To compare two different specimen extraction sites (SES) (off‐midline [OM] vs. supraumbilical) for the incidence of incisional hernias (IHs) and to evaluate different risk factors for their occurrence after robot‐assisted radical prostatectomy.

**Patients and Methods:**

In our clinic, prior to June 2019, SES were supraumbilical; thereafter, they were OM. The primary endpoint was to determine the need for IH repair according to the chosen SES. Additionally, we evaluated possible risk factors. Statistical analysis was performed with the Mann–Whitney *U* test, Chi‐squared, binary and multiple logistic regression analysis, and log‐rank testing.

**Results:**

Data from 914 consecutive patients (April 2018–September 2020) were used for the final analysis; of these, 439 (48%) had a supraumbilical and 475 (52%) an OM SES. After a median follow‐up of 35.0 months, 50 (5.5%) patients developed an IH: 42 (9.5%) from the supraumbilical group and eight (1.7%) from the OM group (*P* < 0.001, odds ratio 5.495; 95% confidence interval 2.42–12.48). Median time to IH repair was 7.9 months: 0.5 in the OM and 10.1 in the supraumbilical group (*P* = 0.026). There were no other associations with clinico‐pathological patient data.

**Conclusion:**

The SES in robot‐assisted radical prostatectomy seems to play an important role in the development of postoperative IHs, with the OM approach potentially offering an advantage. Additionally, IHs appear to occur earlier with an OM than with a supraumbilical approach.

**Abbreviations**
ADT: Androgen deprivation therapy
BMI: Body mass index
CCI: Charlson Comorbidity Index
Chemo: Chemotherapy
COPD: chronic obstructive pulmonary disease
FU: Follow‐up
HT: Hormonal therapy
IHs: Incisional hernias
IQR: Inter quartile range
MIS: Minimally invasive surgery
mo: Months
*N*
: number
OM: Off‐midline
OR: Odds ratio
PC: Prostate cancer
Pt: patients
RALP: robot assisted laparoscopic radical prostatectomy
SESs: Specimen extraction sites
SHT: Secondary hormonal therapy
SU: Supraumbilical

## Introduction

In recent years, robot assisted laparoscopic radical prostatectomy (RALP) has become the standard approach to the surgical therapy of localized prostate cancer (PC), with up to 95% of the nearly 90,000 radical prostatectomies performed in the USA every year now being RALPs [1, 2]. Incisional hernias (IHs) are a common, often underreported complication in both open and minimally invasive surgery (MIS), with incidence rates ranging between 4% and 10% [3]. In MIS, IHs are typically located at specimen extraction sites (SESs), and most cases of IH require surgical repair. This results in not only significant morbidity risks to patients but also significant costs for healthcare systems. Recently, Peyrottes et al. drew attention to this issue by reporting a 1‐year incidence rate of 12.1% for IH after transperitoneal RALP using a supraumbilical (SU) SES [4]. Although literature suggests that the choice of extraction site may influence the occurrence of IH in MIS [5, 6], robust evidence specific to RALP remains sparse.

Therefore, the aim of this study was to evaluate whether there is a difference in the occurrence of IHs in RALP when two different SESs (off‐midline [OM] vs. SU) are used. Additionally, we evaluate further risk factors for IHs in RALP.

## Patients and Methods

### Study Design and Patients

Data from all patients with PC treated at our department were prospectively collected from our PC database; in total 926 consecutive patients underwent RALP between April 2018 and September 2020 (see Fig. 1).

**Fig. 1:**
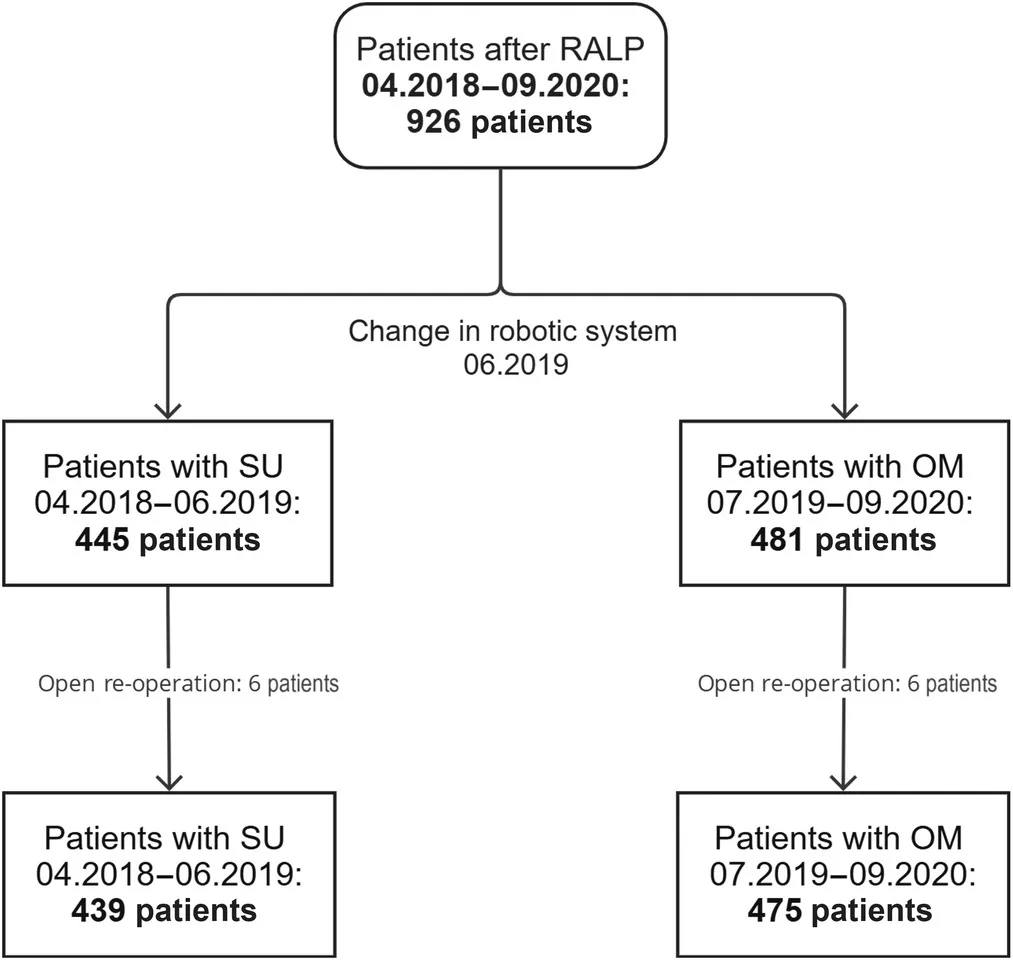
Flow chart. OM, off‐midline specimen extraction site; RALP, robot assisted laparoscopic radical prostatectomy; SU, supraumbilical specimen extraction site.

In June 2019, a new robotic system was introduced into our clinic, and the system was changed from the da Vinci Si to the da Vinci X (both Intuitive Surgical, Palo Alto, CA, USA). Simultaneously, the SESs were changed from an SU to an OM (see Fig. 2).

**Fig. 2:**
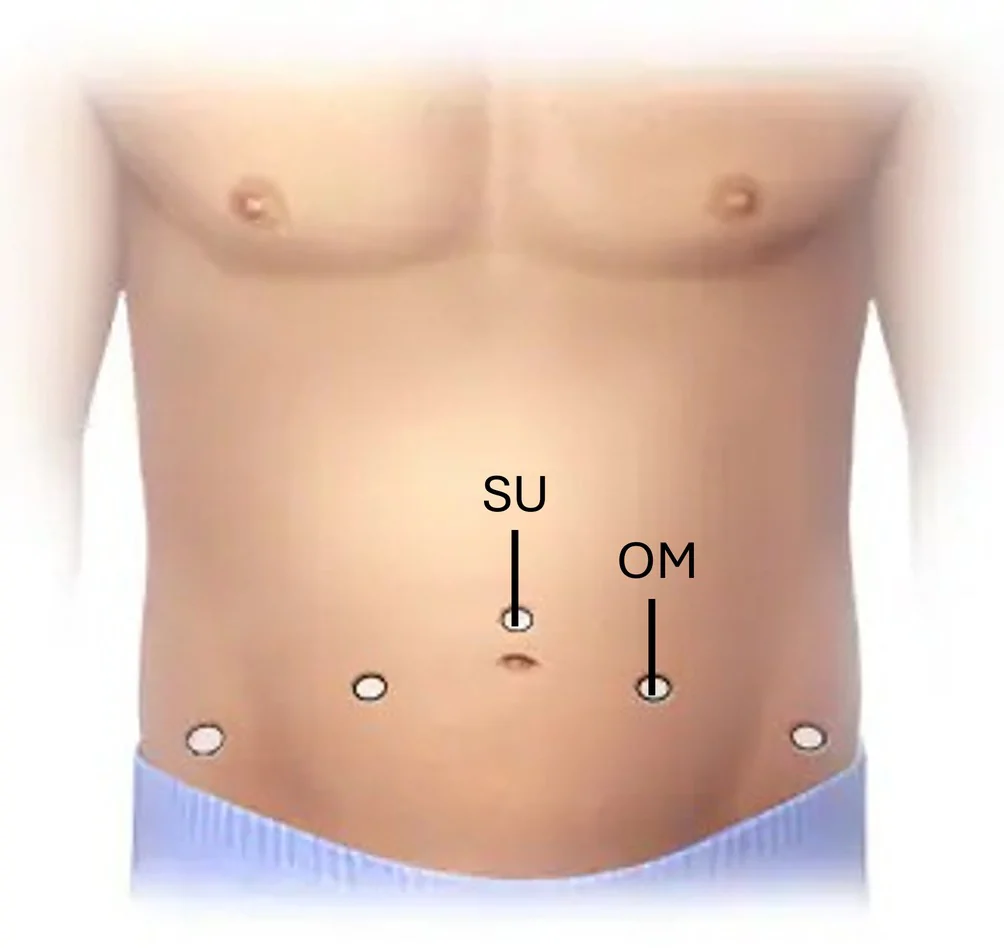
Specimen extraction sites in robot‐assisted laparoscopic radical prostatectomy. OM, off‐midline specimen extraction site; SU, supraumbilical specimen extraction site. Used with permission of Mayo Foundation for Medical Education and Research, all rights reserved; adapted from [7].

Before this system change, 445 patients received an SU SES; after the change, 481 patients received an OM SES. Patients in need of an abdominal laparotomy due to postoperative complications were excluded from this study (six in each cohort), resulting in a total of 439 consecutive patients with an SU SES and 475 consecutive patients with an OM SES for the final data analysis (see Fig. 1). This study was approved by the ethics committee of Saarland, Saarbrücken, Germany (identification number: 10/22).

### Port Placement and Surgical Technique of Specimen Extraction

In all patients who received an SU SES, we placed a 12 mm trocar in the lower left abdomen (assistant trocar; fascial closure at the end of the operation) and an additional 12 mm trocar was placed supraumbilically, later serving as the SES. For the OM SES, we used four 8 mm trocars, including the supraumbilical trocar, and the 12 mm assistant trocar was placed in the OM position, later serving as an SES. All incisions for the later SES (both SU and OM) were performed vertically, including skin, fascia and peritoneum. At the end of the operation when extracting the prostate specimen, the incision was chosen according to the size of the prostate. The console surgeon and/or the bedside physician performed the surgical closure of the fascia (one layer) at the extraction point with a running suture of n°1 Vicryl. All procedures were performed exclusively by highly experienced senior surgeons.

### Follow‐Up

In January 2023, a questionnaire was sent to all the patients to obtain follow‐up (FU) information on the occurrence of IHs, the need for re‐operation, and the cause of the re‐operation (see Table S1).

### Statistical Analyses

The primary endpoint of the study was the need for hernia repair due to the occurrence of IHs. The indication for hernia repair was made based on patients’ symptoms and physical examination. We chose the need for hernia repair as the primary endpoint because of its clinical relevance. The secondary aim was to investigate possible risk factors for the occurrence of IHs. Descriptive statistics are presented as medians (interquartile range [IQR]) or n (%), as an appropriate. Comparisons between the SU and OM groups, as well as between responders and non‐responders to the questionnaire, were performed with the Mann–Whitney *U* or Chi‐squared test, and binary logistic regression analysis was used to investigate the relationship between the binary variable occurrence of IHs and a series of independent variables. Multiple variable logistic regression analysis was performed to further investigate the influence of certain variables on the occurrence of IHs, with a significance cut‐off point set at *P* < 0.05. The variables were chosen by statistical significance in the univariate analysis according to Table S2. Kaplan–Meier analysis was used to evaluate the occurrence of IH in a time‐dependant manner and the Cox proportional hazard model to perform a time‐to‐event analysis. All statistical analyses, including log‐rank and Kaplan–Meier analyses, were conducted with the use of IBM SPSS software (version 28.0; IBM, Armonk, NY, USA).

## Results

### Baseline Characteristics

The median age of our patients was 66 years (IQR 61.0–70.0), and most of the PCs detected were pT2c and pT3a tumours (44% vs. 31%). Intermediate‐risk PCs, defined as Gleason score 7 tumours, were the most common (70%), and median initial prostate‐specific antigen level at diagnosis was 7.9 ng/mL (IQR 1.5–14.3). Most of the patients were pN0 (85%), and 28% had a positive resection margin. Additionally, the median body mass index (BMI) was 27.1 kg/m^2^ (IQR 24.9–29.7), and the median prostate weight was 42.0 g (IQR 33.0–56.0). A total of 14% of the patients received prior antitumor therapies, with these generally being androgen‐deprivation therapy. In total, 11% had diabetes mellitus requiring medical intervention, 3% had chronic obstructive pulmonary disease, 1% had already undergone major abdominal surgery, 26% had already undergone surgery for hernia repair, and 12% experienced hernia treated simultaneously during RALP. In all, 59% of the patients responded to the questionnaire. The median FU was 35 months (IQR 1.0–46.8), and the length of FU was significantly different in the SU group compared with the OM group (SU 49.5 months [IQR 43.2–54.1]; OM 33.7 months [IQR 8.0–36.4], *P* < 0.001). Table 1 demonstrates the baseline characteristics of the entire study collective and of the SU and OM groups. To assess the potential risk of response bias, we performed a comparative analysis between responders and non‐responders to the questionnaire (see Table S1). This analysis revealed significant differences in BMI (responder: lower) and length of follow‐up (responder: longer). Additionally, a non‐significant trend towards a higher occurrence of IH was observed within the responder group.

**Table 1:** Baseline characteristics.

Characteristic	Total, *n* = 914	SU, *n* = 439	OM, *n* = 475	*P*‐value
Age, years	66.0 (61.0–70.0)	66.0 (61.0–70.0)	65.0 (61.0–70.0)	0.250
BMI, kg/m^2^	27.1 (24.9–29.7)	27.0 (24.7–29.6)	27.2 (25.2–29.7)	0.192
CCI				
0–2	439 (48.1)	212 (48.3)	227 (47.9)	0.550
3–5	428 (46.8)	205 (46.7)	223 (46.9)	
>5	47 (5.1)	22 (5.0)	25 (5.2)	
iPSA, ng/mL	7.9 (5.7–12.1)	7.6 (5.6–11.9)	8.0 (5.8–12.4)	0.098**
Preoperative PSA, ng/mL	7.9 (5.4–11.6)	7.9 (5.3–11.6)	8.0 (5.4–11.6)	0.891
Weight of prostate specimen, g	42.4 (33.0–56.0)	42.4 (33.1–55.3)	42.4 (32.7–57.0)	0.872
pT stage				
2a	48 (5.3)	21 (4.8)	27 (5.7)	0.207
2b	2 (0.2)	0 (0)	2 (0.4)	
2c	401 (43.9)	196 (44.7)	205 (43.2)	
3a	287 (31.4)	131 (29.8)	156 (32.8)	
3b	173 (18.9)	91 (20.7)	82 (17.3)	
4	3 (0.3)	0 (0)	3 (0.6)	
pN stage				
x	19 (2.1)	8 (1.8)	11 (2.3)	0.872
0	775 (84.8)	373 (85.0)	402 (84.6)	
1	120 (13.1)	58 (13.2)	62 (13.1)	
R stage				
0	641 (71.9)	306 (71.0)	335 (72.8)	0.544
1	250 (28.1)	125 (29.0)	125 (27.2)	
<pT3	73 (29.2)	36 (28.8)	37 (29.6)	
≥pT3	177 (70.8)	89 (71.2)	88 (70.4)	
Gleason score^†^				
Not possible due to ADT^‡^	68 (7.4)	32 (7.3)	36 (7.6)	0.723
6	59 (6.4)	24 (5.5)	35 (7.4)	
7a	448 (49.0)	220 (50.1)	228 (48.0)	
7b	193 (21.1)	90 (20.5)	103 (21.7)	
8	83 (9.1)	42 (9.6)	41 (8.6)	
9 (4+5)	52 (5.7)	27 (6.1)	24 (5.0)	
9 (5+4)	10 (1.1)	4 (0.9)	6 (1.3)	
10	2 (0.2)	0 (0)	2 (0.4)	
Preoperative antitumor therapies				
No	790 (86.4)	379 (86.4)	411 (86.5)	0.932
Yes	124 (13.6)	60 (13.6)	64 (13.5)	
Standard ADT	105 (84.7)	47 (78.4)	58 (90.6)	
ADT + local therapy^§^	5 (4.0)	2 (3.3)	3 (4.7)	
ADT + SHT	2 (1.6)	2 (3.3)	0 (0)	
ADT + SHT + chemo	3 (2.4)	1 (1.7)	2 (3.1)	
Other^¶^	9 (7.3)	8 (13.3)	1 (1.6)	
Diabetes mellitus				
No	814 (89.1)	398 (90.7)	416 (87.6)	0.136
Yes, oral	79 (8.6)	27 (6.1)	52 (10.9)	
Yes, insulin	21 (2.3)	14 (3.2)	7 (1.5)	
COPD				
No	891 (97.5)	429 (97.7)	462 (97.3)	0.658
Yes	23 (2.5)	10 (2.3)	13 (2.7)	
Preoperative abdominal surgery^††^				
No	468 (51.2)	212 (48.3)	256 (53.9)	0.080^#^
Yes, minor	434 (47.4)	223 (50.8)	210 (44.2)	
Yes, major	13 (1.4)	4 (0.9)	9 (1.9)	
Preoperative hernia repair				
No	676 (74.0)	319 (72.7)	357 (75.2)	0.425
Yes, umbilical	29 (3.2)	12 (2.7)	17 (3.6)	
Yes, inguinal	186 (20.4)	97 (22.1)	89 (18.8)	
Yes, inguinal and umbilical	23 (2.4)	11 (2.5)	12 (2.4)	
Intraoperative hernia repair	113 (12.4)	57 (13.0)	56 (11.8)	0.627
Questionnaire response rate	542 (59.9)	264 (60.1)	278 (58.5)	0.621
Length of follow‐up, months	35.0 (1.0–46.8)	49.5 (43.2–54.1)	33.7 (8.0–36.4)	<0.001*

Data are presented as *n* (%) or median (interquartile range) unless otherwise indicated.

ADT, androgen‐deprivation therapy; BMI, body mass index; CCI, Charlson Comorbidity Index (prostate cancer diagnosis excluded); Chemo, chemotherapy; COPD, chronic obstructive pulmonary disease; iPSA, initial PSA level at diagnosis; OM, off‐midline specimen extraction site; PSA, prostate‐specific antigen; SHT, secondary hormonal therapy; SU, supraumbilical specimen extraction site.

^†^ Prostatectomy specimen.

^‡^ Only patients who received ADT until the day of the operation were counted.

^§^ ADT plus local therapy, comprising patients who received high‐frequency ultrasound, brachytherapy, or cyberknife in combination with ADT.

^¶^ Other comprised five patients who received prior systemic therapies for other malignancies and four patients treated with high‐frequency ultrasound or cyberknife.

^††^ Minor = up to four abdominal surgeries and/or no abdominal laparotomy; major = more than four or complete abdominal laparotomy.

* *P* < 0.05.

# *P* < 0.10.

### Occurrence of Incisional Hernias and Time to Hernia Repair

At a median FU of 35.0 months (IQR 1.0–46.8), 50 patients (5.5%) developed an IH. With 9.5% (*n* = 42) of the SU group and 1.7% (*n* = 8) of the OM group experiencing IHs, the occurrence of IHs in the SU group was significantly higher (*P* < 0.001) (see Table 2 and Fig. 3). The median time to hernia repair was 7.9 months (IQR 0.5–19.1) for the entire cohort. The median time to hernia repair was 10.1 months (IQR 4.0–21.7) in the SU group and 0.5 months (IQR 0.3–2.4) in the OM group, so the time to hernia repair was significantly lower in the OM group (*P* = 0.026) (see Table 2).

**Table 2:** Hernia incidence and time to hernia repair according to study group.

Hernia incidence and time	Total, *n* = 914	SU, *n* = 439	OM, *n* = 475	*P*‐value
Hernias	50 (5.5)	42 (9.5)	8 (1.7)	<0.001*
Months to hernia repair	7.9 (0.5–19.1)	10.1 (4.0–21.7)	0.5 (0.3–2.4)	0.026*

Data are presented as *n* (%) or median (interquartile range) unless otherwise indicated.

OM, off‐midline specimen extraction site; SU, supraumbilical specimen extraction site.

* *P* < 0.05.

**Fig. 3:**
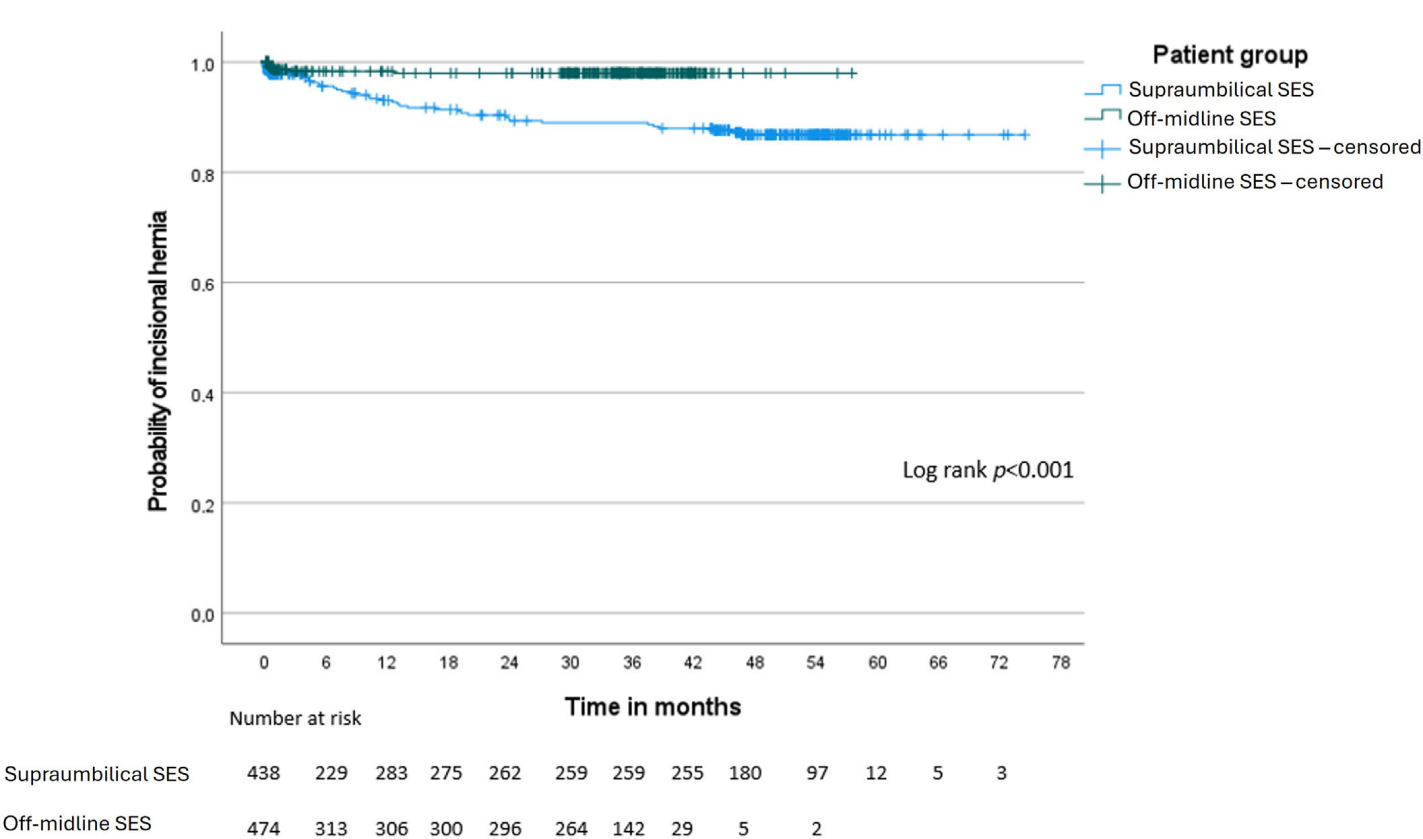
Kaplan–Meier analysis of the probability of incisional hernias. SES, specimen extraction site.

In the Cox proportional hazard model for time‐to‐event analysis, developing an IH was significantly more likely in patients with an SU than in those with an OM SES (hazard ratio [HR] 5.918, *P* < 0.001). Additionally, patients who answered the questionnaire were significantly more likely to have developed an IH (HR 2.604, *P* = 0.006). After surgical repair of IHs, the failure rate was 4/50 (8%) for the whole study population, with two (4%) each in the SU and OM groups.

### Risk Factors for Incisional Hernias

When evaluating possible risk factors for IHs, the SES was the only independent risk factor for IH occurrence, as indicated via univariate and multivariate analysis (see Table S2 and Table 3). Undergoing an SU SES was associated with a 5.495 elevated risk of developing an IH. There was no association with BMI, prostate weight, diabetes mellitus, or other important risk factors associated with general hernia occurrence (see Table S2).

**Table 3:** Multivariable, binary logistic regression analysis of possible risk factors for incisional hernias.

Risk factor	*P*‐value	OR	95% CI
SU	<0.001*	5.495	2.419–12.484
Follow‐up, months	0.433	1.009	0.986–1.033
Response rate	0.658	1.269	0.442–3.644

CI, confidence interval; OR, odds ratio; SU, supraumbilical specimen extraction site.

* *P* < 0.05.

## Discussion

According to our study, SU SES is independently associated with the occurrence of IHs: 9.5% of patients developed an IH with an SU SES compared with 1.7% with an OM SES. We found no other risk factors in univariate or multivariate analysis, that predicted IHs. We also found that OM hernias occurred significantly earlier than SU hernias (0.5 months vs. 10.1 months, respectively).

The risk of IHs is influenced by the surgical technique, with MIS in general reducing the risk compared with open surgery (from 10.1% to 4.3%) [3, 8]. However, two large register studies that investigated IHs in radical prostatectomy presented a different picture [9, 10]. Carlsson et al. conducted a Surveillance, Epidemiology, and End Results (SEER) database analysis of 9994 men and reported a more‐than‐threefold increased risk for IH repair in the MIS group (HR 3.39; *P* < 0.0001), with a frequency of 5.3% (median FU 3.1 years) compared with 1.9% in a comparative open surgery group (median FU 4.4 years). The authors suggested that the SU SES in the MIS group could explain these findings [9]. A Swedish study of 19,743 men by Hermann et al. from the National Prostate Cancer Register also found a significantly higher incidence of IHs with the MIS approach (2.3% MIS vs 1.4% open) [10]. In our study on IHs in RALP, we found an overall IH incidence rate of 5.5%, similar to the results reported by Carlsson et al., albeit slightly higher than the results of Hermann et al. for their MIS groups. We also found a significant difference in the rate of IHs according to SES, with an IH rate of 1.7% with an OM approach versus 9.5% with an SU approach, supporting the hypothesis that the SU SES plays an important role.

The frequency of IHs according to SES is best investigated in MIS colorectal surgery, as demonstrated by Benlice et al. in a group of 2148 patients who underwent laparoscopic colorectal surgery. These authors discovered that, compared with the use of Pfannenstiel as the SES (0.9%), the independent factors associated with IHs were (among others) the periumbilical midline SES (12.6%, HR 12.7), increased BMI (HR 1.23), synchronous port‐site hernias (HR 3.66), and postoperative superficial surgical‐site infection (HR 2.11) [5]. These observations were strengthened by Lee et al., who conducted a meta‐analysis in laparoscopic colorectal surgery. That study found a pooled incidence of IHs (10.6% in midline, 3.7% in transverse, and 0.9% in Pfannenstiel SESs) demonstrating a significantly higher IH incidence for the midline SES group (weighted odds ratio 4.1; 95% confidence interval 2.0–8.3). Therefore, the updated guidelines of the European and American Hernia Societies recommend avoiding midline incisions for specimen extractions to reduce the risk of IHs [3].

However, in terms of RALP, there is less evidence available that compares different SESs. In a small retrospective series, Zukivskyj et al. found a lower rate of IHs with a Pfannenstiel SES (6.7%) than with an SU SES (12.7%); however, no statistical significance was demonstrated [11]. Recently, in a prospectively maintained database of 1465 patients, van Horn et al. compared SU SESs and Pfannenstiel SESs and found a significant reduction in IHs with a Pfannenstiel SES (0.6% vs 7.4%), supporting the results of Zukivskyj et al. [12] Except for a higher prevalence in Hispanic patients, they found no other associations with a lower IH incidence via multivariate regression analysis. Seveso et al. conducted a study similar to ours, performing a retrospective analysis of 800 patients after RALPs performed between 2005 and 2014, with a follow‐up of at least 3 years (median and IQR not reported), and comparing the IH rates between SU and OM SESs [13]. In contrast to our results, they did not find a significant difference in IH occurrence (SU 5%; OM 4.5%). However, they conducted office visits, and their primary endpoint was clinically evident hernia occurrence upon Valsalva manoeuvre, whereas we used a standardized questionnaire for FU and surgical repairs or re‐operations as our primary endpoint. When comparing our primary endpoint to the results from Seveso et al., their SU group had a 2.5% (*n* = 10/400) hernia rate compared with the 1.3% (*n* = 5/400) hernia rate in their OM group. Additionally, they did not find a difference in the time to hernia occurrence (20.2 months for SU; 18.2 months for OM); in our study, time to hernia occurrence was significantly earlier with OM (0.5 months vs. SU 10.1 months). However, Seveso et al. used different closure techniques at SU sites (single layer of n°1 Vicryl, interrupted) and OM sites (two layers with n°0 Vicryl, interrupted), whereas we used a single layer of n°1 Vicryl, continuous technique, in both cases. Furthermore, slightly different da Vinci setups were used. Recently, Peyrottes et al. reported a 1‐year IH prevalence of 12.1% in transperitoneal RALP with an SU SES, thus corroborating our findings of an IH incidence of 9.5% for the SU cohort [4].

Beck et al. reported that, using a transverse skin and fascial incision instead of a vertical one at the SU SES significantly decreased the risk of IH from 5.3% to 0.6% [14]. However, Gaffney et al. did not find a significant difference between transverse and vertical fascial incisions (transverse 21% vs. vertical 19%, *P* = 0.500) when testing this hypothesis in a prospective randomized trial [15]. It is worth noting that the overall incidence of 20% reported by Gaffney et al. is quite high compared with other reports; nevertheless, this constitutes the highest level in existing evidence so far. Recently, Adiyat et al. published a series of retzius‐sparing RALP in more than 1000 consecutive patients [16]. They reported a significant reduction of IHs after they changed their SES from an SU to a Pfannenstiel incision: 7.7% with SU compared with 0% with Pfannenstiel.

Our results stress the importance of adherence to the recommendations of the Hernia Societies, which is to avoid an SU SES, also in terms of RALP. A possible biological explanation is that the SU SES has a weaker fascial component because it lies between the rectus muscles, whereas the off‐midline SES lies within the rectus muscle and thus represents a stronger part of the abdominal wall. One open question remains, which is whether a Pfannenstiel SES or OM SES is preferred. Thus far, no comparative studies on the two have been conducted.

Several patient‐related risk factors for IHs, identified by the European Hernia Society, include diabetes, obesity, smoking, and immunosuppressive therapy [17]. In our study, we found no risk factors other than the SES to be independently associated with IH occurrence. Other studies on the risk of IH in RALP found associations with age, prostate volume, lymph node dissection, and BMI, with prostate volume being the most consistently reported [4, 13, 14, 18]. However, not all of these studies performed multiple testing, and the study designs differed widely. Additionally, factors such as BMI and prostate volume are subject to geographical variations, which might explain the lack of association observed in our patient cohort. Diabetes mellitus may be underestimated in our study because of its retrospective design and the exclusive capture of pharmacologically treated cases.

We found a significantly earlier occurrence of OM IHs compared with SU IHs, with a median time to IH occurrence of 0.5 months in the OM group and 10.1 months in the SU group. We therefore hypothesize that an OM IH more often occurs during or shortly after the hospital stay, thus becoming clinically evident earlier than an SU IH. However, possible explanations for this observation remain obscure. Using an SU SES, Fuller et al. found a 4.6% incidence of IHs, with a mean time to diagnosis of 14.8 months [18]. More recently, Timm et al. found an IH rate of 9.7% and a mean time to diagnosis of 11.8 months with an SU SES [19]. The IH rate and time to hernia diagnosis were very similar to ours: 9.5% in the SU population; median time to hernia repair 10.1 months.

Nevertheless, our study contained several limitations. This study potentially involved lead‐time bias, given that the SU patients were all operated earlier than the OM patients. Consequently, the FU time was significantly longer in the SU group. Therefore, we included the time to follow‐up in our multivariate analysis as well as a Cox proportional hazards model for time‐to‐event analysis. Additionally, the patients were not included via prospective randomization, and data were collected retrospectively; however, questionnaire data were collected prospectively. The retrospective nature of the study means that wound infections were not documented. Moreover, hernia assessment was conducted via standardized questionnaires and not via clinical patient examination: As supported by our comparison between questionnaire responders and non‐responders, patients who replied may have been more likely to report a hernia. However, their length of follow‐up was longer, suggesting that the true rate of hernia occurrence in the non‐responder group might be underestimated because of the shorter observation times. Finally, the change of the robotic system might be a source of bias.

## Conclusion

The SES in RALP may play a significant role in the development of postoperative IHs, with an up to 5.5‐fold increased risk of developing an IH when an SU approach is used. Additionally, IHs seem to occur earlier when an OM approach is used. According to our findings, OM SES may be preferred over SU SES for RALP.

## Disclosure of Interests

None declared.

## Funding

The authors have nothing to report.

## Supporting information


**Table S1.** Comparison between responders and non‐responders to the questionnaire.
**Table S2.** Univariate analysis of possible risk factors for incisional hernias.

## Data Availability

The data that support the findings of this study are available from the corresponding author upon reasonable request.
