# Supplementary material for: Off‐midline versus supraumbilical specimen extraction sites in robot‐assisted radical prostatectomy
Source: BJU Int. 2026 Aug 5;138(5):886–93. doi: 10.1111/bju.70400 (PMC13655200; doi:10.1111/bju.70400)
Supplement: Supplementary file 1 [file BJU-138-886-s001.docx]

|  | Responders n= 542 | Non-responders  n= 372 | p-value |
| --- | --- | --- | --- |
| Age in years, median (IQR) | 66 (9) | 65 (9) | 0.077 |
| BMI in kg/m², median (IQR) | 26.9 (4.5) | 27.5 (5.5) | **0.020*** |
| CCI (>5) (%) | 20 (3.7) | 27 (7.25) | 0.079 |
| iPSA, median (IQR) | 7.70 (5.95) | 8.00 (7.4) | 0.180 |
| Preoperative PSA, median (IQR) | 7.81 (5.93) | 8.05 (7.61) | 0.220 |
| Weight of prostate specimen (g), median (IQR) | 42.9 (22.4) | 42.0 (23.7) | 0.228 |
| pT-stage (>pT2), (%) | 263 (48.5) | 200 (53.4) | 0.280 |
| pN-stage (pN1), (%) | 74 (13.6) | 46 (13.4) | 0.725 |
| R-Stage (R1), (%) | 155 (28.6) | 95 (25.5) | 0.339 |
| Gleason Score (>7), (%) | 81 (14.9) | 65 (17.5) | 0.744 |
| Preop. ADT (yes), (%) | 70 (12.9) | 54 (14.5) | 0.487 |
| Diabetes mellitus (yes), (%) | 55 (10.1) | 45 (12.0) | 0.354 |
| COPD (yes), (%) | 10 (1.8) | 14 (3.8) | 0.075 |
| Preoperative abdominal surgery (yes), (%) | 277 (51.1) | 169 (45.4) | 0.224 |
| Preoperative hernia repair (yes), (%) | 145 (26.7) | 94 (25.2) | 0.616 |
| Intraoperative hernia(yes), (%) | 61 (11.2) | 52 (14.0) | 0.219 |
| Extraction site  SUI  OMI | 264 (48.7)  278 (51.3) | 175 (47.0)  197 (53.0) | 0.621 |
| Follow Up in months, median (IQR) | 42.5 (15.7) | 0.3(4.7) | **<0.001*** |
| Incisional hernia rate (yes), (%) | 36 (6.6) | 14 (3.8) | **0.060 ^ꝉ^** |

Table S1: Comparison between responders and non-responders to the questionnaire.

*n = number; IQR = interquartile range; BMI = body mass index; CCI = Charlson Comorbidity Index; iPSA = initial PSA level at diagnosis; ADT = androgen deprivation therapy; COPD = chronic obstructive pulmonary disease; SUI = supraumbilical specimen extraction site; OM = off-midline specimen extraction site; *p< 0.05;* ^ꝉ^*p<0.10*

|  | Incisional hernia n= 50 | No incisional hernia  n= 864 | p-value |
| --- | --- | --- | --- |
| Age in years, median (IQR) | 67.5 (61.7-72.2) | 66.0 (61.0-70.0) | 0.139 |
| BMI in kg/m², median (IQR) | 27.7 (25.2-30.2) | 27.1 (24.9-29.6) | 0.345 |
| CCI > 5, % | 1 (2.0) | 46 (5.3) | 0.306 |
| iPSA, median (IQR) | 7.2 (5.3-14.0) | 7.9 (5.7-12.0) | 0.787 |
| Preoperative PSA, median (IQR) | 7.5 (4.9-12.6) | 7.9 (5.4-11.6) | 0.717 |
| Weight of prostate specimen (g),  median (IQR) | 43.4 (34.2-54.6) | 42.3 (39.9-56.0) | 0.745 |
| pT-stage (>pT2), % | 28 (56.0) | 435 (50.3) | 0.664 |
| pN-stage (pN1), % | 6 (12.0) | 114 (13.2) | 0.808 |
| R-Stage (R1), % | 13 (26.0) | 237 (27.4) | 0.739 |
| Gleason Score (>7), % | 6 (12.0) | 140 (16.2) | 0.514 |
| Preop. ADT (yes), (%) | 10 (20) | 114 (13.2) | 0.172 |
| Diabetes mellitus (yes), (%) | 6 (12.0) | 94 (10.9) | 0.805 |
| COPD (yes), (%) | 3 (6.0) | 20 (2.3) | 0.106 |
| Preoperative abdominal surgery (yes), (%) | 26 (52.0) | 420 (48.6) | 0.858 |
| Preoperative hernia repair (yes), (%) | 12 (24.0) | 226 (26.2) | 0.735 |
| Intraoperative hernia | 6 (12.0) | 107 (12.4) | 0.936 |
| Extraction site  SU  OM | 42 (84.0)  8 (16.0) | 397 (45.9)  467 (54.1) | **<0.001*** |
| Follow-up in months, median (IQR) | 46.2 (31.9-53.6) | 34.7 (0.8-46.4) | **<0.001*** |
| Response rate (yes), (%) | 36 (72.0) | 506 (58.6) | **0.060 ^ꝉ^** |

Table S2: Univariate analysis of possible risk factors for incisional hernias.

*n = number; IQR = interquartile range; BMI = body mass index; CCI = Charlson Comorbidity Index; iPSA = initial PSA level at diagnosis; ADT = androgen deprivation therapy; COPD = chronic obstructive pulmonary disease; SUI = supraumbilical specimen extraction site; OM = off-midline specimen extraction site; *p< 0.05;* ^ꝉ^*p<0.10*
